# LAPAROSCOPIC REDO FUNDOPLICATION ALONE, REDO NISSEN FUNDOPLICATION, OR TOUPET FUNDOPLICATION COMBINED WITH ROUX-EN-Y DISTAL GASTRECTOMY FOR TREATMENT OF FAILED NISSEN FUNDOPLICATION

**DOI:** 10.1590/0102-672020220002e1678

**Published:** 2022-09-09

**Authors:** Italo Braghetto, Owen Korn, Manuel Figueroa-Giralt, Catalina Valenzuela, Ana Maria Burgos, Carlos Mandiola, Camila Sotomayor, Eduardo Villa

**Affiliations:** 1 University of Chile, Faculty of Medicine, Hospital “Dr. José J. Aguirre”, Department of Surgery – Santiago, Chile.

**Keywords:** Gastroesophageal Reflux, Recurrence, Fundoplication, Laparoscopy, Gastrectomy, Refluxo Gastroesofágico, Recidiva, Fundoplicatura, Laparoscopia, Gastrectomia

## Abstract

**BACKGROUND:**

Laparoscopic Nissen fundoplication fails to control the gastroesophageal reflux in almost 15% of patients, and most of them must be reoperated due to postoperative symptoms. Different surgical options have been suggested.

**AIMS:**

This study aimed to present the postoperative outcomes of patients submitted to three different procedures: redo laparoscopic Nissen fundoplication alone (Group A), redo laparoscopic Nissen fundoplication combined with distal gastrectomy (Group B), or conversion to laparoscopic Toupet combined with distal gastrectomy with Roux-en-Y gastrojejunostomy (Group C).

**METHODS:**

This is a prospective study involving 77 patients who were submitted initially to laparoscopic Nissen fundoplication and presented recurrence of gastroesophageal reflux after the operation. They were evaluated before and after the reoperation with clinical questionnaire and objective functional studies. After reestablishing the anatomy of the esophagogastric junction, a surgery was performed. None of the patients were lost during follow-up.

**RESULTS:**

Persistent symptoms were observed more frequently in Group A or B patients, including wrap stricture, intrathoracic wrap, or twisted fundoplication. In Group C, recurrent symptoms associated with this anatomic alteration were infrequently observed. Incompetent lower esophageal sphincter was confirmed in 57.7% of patients included in Group A, compared to 17.2% after Nissen and distal gastrectomy and 26% after Toupet procedure plus distal gastrectomy. In Group C, despite the high percentage of patients with incompetent lower esophageal sphincter, 8.7% had abnormal acid reflux after surgery.

**CONCLUSIONS:**

Nissen and Toupet procedures combined with Roux-en-Y distal gastrectomy are safe and effective for the management of failed Nissen fundoplication. However, Toupet technique is preferable for patients suffering from mainly dysphagia and pain.

## RESUMO

**RACIONAL:**

A fundoplicatura de Nissen laparoscópica falha em controlar o refluxo gastroesofágico em quase 15% dos pacientes e a maioria deles deve ser reoperada devido aos sintomas. Diferentes técnicas cirúrgicas têm sido sugeridas.

**OBJETIVOS:**

Apresentar os resultados pós-operatórios de pacientes submetidos a 3 procedimentos diferentes: Reoperação e fundoplicatura de Nissen laparoscópica apenas (Grupo A), reoperação e fundoplicatura de Nissen laparoscópica combinado com gastrectomia distal (Grupo B) ou reoperação e conversão para Toupet laparoscópico combinado com gastrectomia distal e reconstrução em Y-Roux (Grupo C).

**MÉTODOS:**

estudo prospectivo incluindo 77 pacientes submetidos inicialmente a fundoplicatura de Nissen laparoscópica que apresentaram recidiva do refluxo gastroesofágico após a operação. Eles foram avaliados antes e após a reoperação com questionário clínico e estudos funcionais específicos. Reestabelecida a anatomia da junção esôfago-gástrica, procedeu-se às referidas cirurgias. Nenhum paciente perdeu seguimento.

**RESULTADOS:**

Os sintomas persistentes foram observados com maior frequência nos pacientes dos Grupos A ou B, associados à estenose da fundoplicatura, fundoplicatura intratorácico ou fundoplicatura torcida. No Grupo C, os sintomas recorrentes associados a essa alteração anatômica foram observados com pouca frequência. Esfincter esofágico inferior incompetente foi confirmado em 57,7% dos pacientes pertencentes ao Grupo A em comparação com 17,2% após fundoplicatura de Nissen laparoscópica combinado com gastrectomia distal, e 26% após a Toupet laparoscópico combinado com gastrectomia distal e reconstrução em Y-Roux. Nesse grupo, apesar do alto percentual de pacientes com esfincter esofágico inferior incompetente, 8,7% apresentaram refluxo ácido anormal após a cirurgia.

**CONCLUSÕES:**

As técnicas de Nissen ou Toupet combinados com a gastrectomia distal em Y de Roux são seguras e eficazes para o manejo da falha da fundoplicatura a Nissen. A técnica de Toupet é preferível para pacientes que sofrem principalmente com disfagia e dor.

## INTRODUCTION

Laparoscopic Nissen fundoplication (LNF) is the surgical technique of choice to treat patients with gastroesophageal reflux disease (GERD). However, it fails in almost 15% of patients and most of them must be reoperated to treat postoperative symptoms^4,14,19,24,28,40^. The main clinical manifestation is the combination of heartburn, dysphagia, and retrosternal pain. Revisional surgery should be performed in these cases^5,21,22,38,40^.

The options to treat this failure are as follows:

- redo Nissen procedure alone;
- distal gastrectomy alone; or
- combination of redo fundoplication plus distal gastrectomy with Roux-en-Y gastrojejunostomy.

The objective of this prospective study was to report the early- and middle-term postoperative outcomes of patients undergoing a revisional surgery comparing these procedures. Preoperative and postoperative symptoms, endoscopy, radiology, manometry, and 24-h pH monitoring studies were analyzed.

## METHODS

### Patients studied

This study includes a cohort of 78 (13.9%) patients (23 men and 54 women), with a mean age of 45.9 years (range 34–61), who were previously submitted to Nissen fundoplication for GERD.

The study patients were first operated on 5.72±1.69 years before (range 1–8). They started to present recurrence of reflux symptoms, 3.59±1.81 years after the primary operation. They had to be reoperated due to unsatisfactory response to medical treatment and the presence of esophagitis. Symptoms and objective studies were performed. They were submitted to three different procedures:

#### Group A:

Redo Nissen fundoplication (LNFDG) alone performed in 26 patients, due to recurrent reflux symptom and severe esophagitis despite medical treatment

#### Group B:

Redo laparoscopic Nissen fundoplication combined with Roux-en-Y distal gastrectomy (LTFDG), performed in 29 patients because they presented reflux symptoms associated with Barrett’s esophagus (BE), due to long history of recurrent reflux symptoms. Because our Hospital is a center of reference, most patients with BE are sent to us for definitive treatment.

#### Group C:

Laparoscopic conversion to Toupet fundoplication combined with Roux-en-Y distal gastrectomy (LTFDG), because they presented mainly chest pain and dysphagia (n=23).

Patients included in this study had a mean body mass index (BMI) of 26.7±4.5 kg/m^2^ without differences between the patients included in each group.

### Inclusion criteria

- patients previously submitted to Nissen fundoplication;
- symptomatic patients;
- failed Nissen fundoplication with anatomical deformities; and
- nonresponders to medical treatment.

### Exclusion criteria

- patients presenting large hiatal hernia;
- asymptomatic patients after fundoplication;
- patients submitted to other upper esophagogastric surgery; and
- obese patients.

All patients gave their informed written consent to be included in this study.

### Preoperative study

#### Symptoms:

A face-to-face interview was conducted to evaluate for the presence of heartburn, dysphagia, and retrosternal pain, according to the DeMeester’s score^20^.

#### Endoscopic evaluation:

This standardized procedure was performed using CV 190 Olympus flexible gastroscope after a 12-h fast and pharyngeal anesthesia with lidocaine and Midazolam® intravenous injection. The examination was done to inspect the squamous-columnar junction establishing the presence of erosive esophagitis defined according to the Los Angeles classification and to detect the presence of cardia dilatation or hiatal hernia using the Hill classification. The presence of BE was defined and classified using the Praga definition. Biopsies were taken in order to have histological confirmation of the presence of esophagitis or intestinal metaplasia^2,23^.

#### Radiologic evaluation:

Patients were submitted to a barium swallow examination in order to evaluate the anatomic aspect of the fundoplication, defining reflux presence when the radiologist confirms ascending barium content to the upper esophagus and evaluating esophageal emptying through esophagogastric junction (EGJ).

#### Manometric studies:

A standard or high-resolution manometry was performed after 12 h fast and before the pH monitoring. The resting pressure, abdominal length of the lower esophageal sphincter (LES), and amplitude of distal esophageal contractile waves were measured^14,15^.

#### 24-h pH monitoring:

This was carried out after a 12-h fast by introducing a catheter through the nose into the stomach, after having stopped proton-pump inhibitors (PPIs) treatment 8 days before the study. The tip was placed 5-cm proximal to the upper border of the LES^30^.

#### Histologic analysis:

During all endoscopic procedures, at least eight biopsy samples were taken from the distal esophagus above and below the Z-line. All samples were immediately placed in a 10% formalin solution and sent to histologic examination. After standard processing, all units were stained with hematoxylin-eosin and Alcian blue at pH of 2.5, searching for the presence of intestinal metaplasia. An expert pathologist examined the epithelium^16^.

#### Clinical outcome:

The postoperative early complications observed after the operation were defined using Clavien-Dindo score and late symptoms were classified according to the Visick score^12,42^.

#### Follow-up:

Clinical control was assessed with the same presented questionnaire in order to determine the presence of moderate or severe recurrent symptoms according to the DeMeester’s reflux symptoms score. Objective studies were repeated 6–12 months on each patient after their primary intervention. The mean follow-up age is 4.3±0.95 years (range 2–8).

#### Statistical analysis:

The analysis was performed using chi-square test. GraphPad program was applied to each group for comparison. To assess significance, a Fisher’s exact test and Student’s t-test were performed based on the variable distribution. A statistical significance was defined as p<0.05.

#### Ethics statements:

All patients gave their written informed consent to be included in this study. All procedures in human participants were in accordance with the Institution and Ministerial Committee and with the 1961 Helsinki Declaration and its later amendments or comparable ethical standards.

#### Surgical procedure:

**Figure 1:**
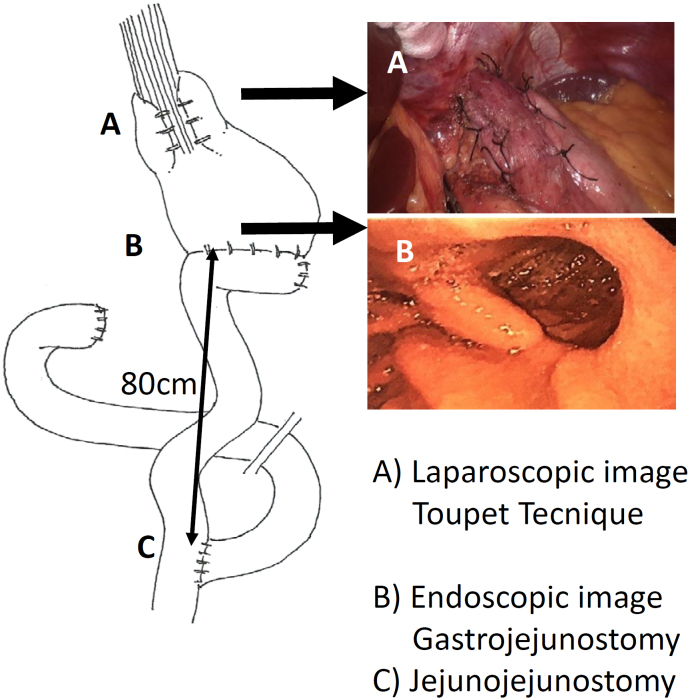
Surgical technique for laparoscopic Toupet fundoplication combined with distal gastrectomy and Roux-en-Y gastrojejunostomy.

The main steps for the surgical procedures are as follows:

- Patients who underwent surgery in the past were necessary to perform adhesiolysis of attachments of the inferior face of the liver to the gastrohepatic ligament and upper part of the stomach in order to identify the EGJ, hiatal crura, and distal esophagus. This can be done using Harmonic scalpel HD®1000i (Ethicon Johnson&Johnson Medical Devices) or monopolar hook.
- Fundoplication is disarmed completely, avoiding damage of gastric or esophageal wall.
- Redo Nissen or Toupet procedures were performed^22,25,40,47^.
- Hiatus closure with 2-3 stiches with non-absorbable suture is necessary (Silk 00 SH CO12D, Ethicon, Johnson&Johnson Medical Devices).
- Distal gastrectomy: The gastroepiploic vessels of the greater curvature of the stomach are divided using Harmonic scalpel (HD®1000i, Ethicon, Johnson&Johnson Medical Devices) until 1 cm distal to the pylorus, right gastric artery is dissected by an anterior approach and divided with Harmonic scalpel. The duodenal bulb is transected 1 cm distal to the pylorus using Endogia Tristaple^TM^ articulating Reload 60-mm purple cartridge (Covidien, Medtronic). The lesser curvature of the stomach is dissected in order to prepare the gastric transection, which is performed horizontally first and then obliquely using Endogia Tristaple^TM^ articulating Reload 60-mm purple cartridge.
- Roux-en-Y gastrojejunostomy: Using a 30-mm Endogia Tristaple^TM^ white cartridge (closing at 2.5 mm), the angles of the suture are reenforced and the orifice closed by a running suture with 00 absorbable V-loc® (Covidien, Medtronic). To avoid stricture at the anastomosis, the suture is placed over the bougie 36F passed distally to the anastomosis. A latero-lateral jejunojejunostomy 80 cm distally is performed using a 45-mm Endogia Tristaple^TM^ white cartridge (Figure 1).

## RESULTS

Early and late postoperative complications of each group are shown in Table 1. Few early complications were observed: 11.5% in Group A, 20.7% in Group B, and 21.7% in Group C. One (3.5%) patient in Group B died due to a nonsurgical complication.

**Table 1:** Early and late postoperative complications after laparoscopic reoperation due to failure after Nissen fundoplication according to the procedure employed.

		A (n=26)	B (n=29)	C (n=23)
EARLY COMPLICATIONS				
	Hemoperitoneum	1 (3.8%) (IIIb)*	–	–
	Pneumonia	–	2 (7.2%) (IIIa)*	–
	Esophageal perforation	–	1 (3.5% (IIIb)*	–
	Bowel obstruction	–	1 (3.5%) (IIIb)*	–
	Subphrenic abscess	2 (7.6%) (IIIb)*		–
	Intraluminal bleeding	–	1 (3.5%) (IIIa)*	1 (4.3%) (IIIa)*
	Vascular brain stroke	–	1 (3.5%) (V)*	–
	Gastric retention	–	–	2 (8.6%) (IIIa)*
	Perigastric collection	–	–	1 (4.3%) (IIIa)*
	Paralytic ileus	–	–	1 (4.3%) (IIIa)*
TOTAL MORTALITY		3 (11.5%)	6 (20.7%)	5 (21.7%)
LATE COMPLICATIONS		0	1 (3.5%)	0
LATE COMPLICATIONS				
	Diarrhea	–	6 (20.7%) (II)**	4 (17.4%) (II)
	Dumping	–	2 (6.9%) (II)**	
	Weight loss	–	3 (10.3%) (II)**	3 (13.1%) (II)**
	Anastomotic ulcer	–	–	1 (4.3%) (II)**
	Anemia	–	1 (3.5%) (II)	1 (4.3%) (II)
	Dysphagia (persistent)	6 (23.1%)	8 (27.5%)	1 (4.3%)

* Clavien-Dindo classification

** Visick score.

None of the patients were lost during follow-up. Persistent dysphagia was observed more frequently in patients submitted to redo Nissen fundoplication. Six patients in Group A must be reoperated due to severe persistent dysphagia and nonresponders to periodic endoscopic dilatation. Other eight patients presented dysphagia after Nissen with Roux-en-Y distal gastrectomy. Only one patient presented this complication after Toupet fundoplication (Table 2).

**Table 2:** Gastroesophageal reflux symptoms before and after laparoscopic reoperation for treatment of failed primary Nissen fundoplication, according to the procedure employed.

			A (n=26)	B (n=29)	C (n=23)
Symptoms					
	Heartburn	Pre-operative	26 (100%)	29 (100%)	23 (100%)
		Postoperative	5 (19.2%)	2 (6.8%)	2 (8.6%)
(A vs. B and C=p<0.001)					
	Regurgitation	Pre-operative	26 (100%)	28 (100%)	23 (100%)
		Postoperative	3 (11.5%)	2 (6.8%	0
(A vs. other groups=p<0.001)					
	Dysphagia	Pre-operative	2 (7.6%)	4 (13.7%)	18 (78.3%)
		Postoperative	6* (23.1%)	8 (27.5%)**	1 (4.3%)**
(A vs. B and C=p<0.001)					
	Chest pain	Pre-operative	8 (30.7%)	0	15 (65.2%)
		Postoperative	1 (3.8%)*	0	1 (4.3%)
	Respiratory	Pre-operative	1 (3.8%)	0	0
		Postoperative	1 (3.8%)	0	0

* Seven patients submitted to second reoperation: five to conversion to redo Nissen with distal gastrectomy and two to Toupet with distal gastrectomy

** Submitted to endoscopic dilatation with Savary bougie.

Other late symptoms occurred in the group of patients submitted to distal gastrectomy, including weight loss, diarrhea, and dumping, catalogued as Visick II. Table 3 shows the preoperative and postoperative wrap characteristics. Preoperatively, wrap disruption, slipped Nissen, and intrathoracic wrap were the most frequent causes of symptoms and for indication for reoperation.

**Table 3:** Radiological assessment with barium swallow before and after laparoscopic reoperation for the treatment of failed primary Nissen fundoplication, according to the procedure employed.

			A (n=26)	B (n=29)	C (n=23)
Radiological findings					
	Wrap disruption	Pre-operative	21 (80.7%)	24 (82.7%)	0
		Postoperative	0	0	0
	Wrap stricture	Pre-operative	0 (100%)	0	7
		Postoperative	5 (19.2%)*	6 (20.7%)*	1 (8.7%)
(A, B vs. C= p<0.001)					
Paraesophageal sliding					
	Hernia	Pre-operative	0	0	4
		Postoperative	0	0	0
	Slipped Nissen	Pre-operative	2 (7.7%)	1 (3.5%)	2 (8.6%)
		Postoperative	0	0	0
	Intrathoracic wrap	Pre-operative	3 (11.5%)	0	8 (34.7%)
		Postoperative	3 (11.5%)	0	0
	Twist or bilobed	Pre-operative	0	0	2 (8.6%)
		Postoperative	4 (15.4%)**	0	0
Reoperated due to refailure			7 (26%)**	0	0

* Endoscopic dilatation not reoperated

** Seven patients submitted to second reoperation: five to conversion to redo Nissen with distal gastrectomy and two to Toupet with distal gastrectomy.

**Figure 2:**
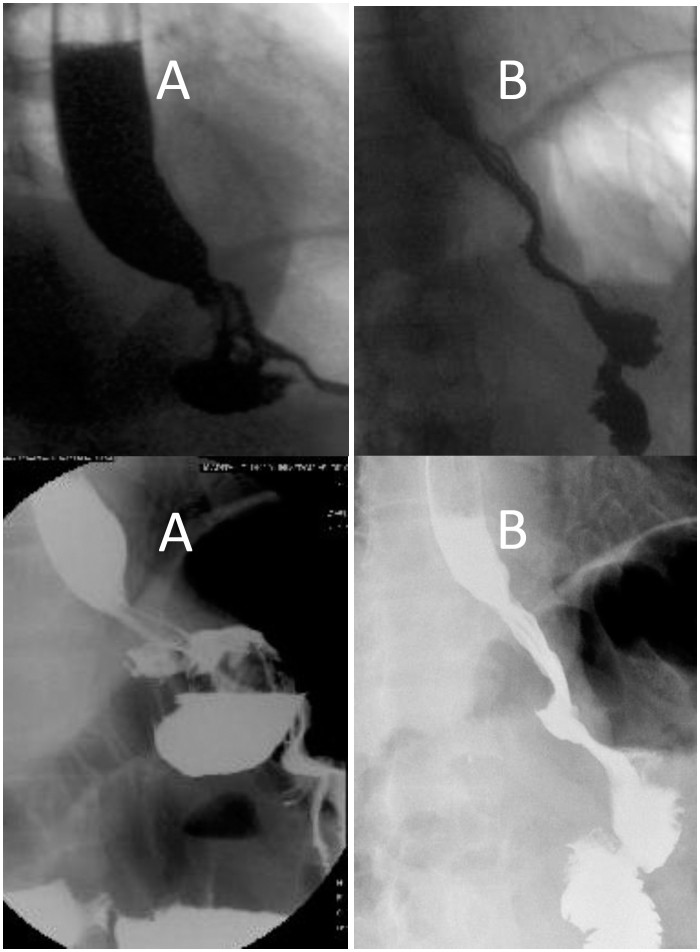
Radiological preoperative and postoperative barium swallow: (A) Asymmetric Nissen fundoplication and stricture at esophagogastric junction; (B) postoperative control of Toupet fundoplication without retention, and gastric emptying through the gastrojejunal anastomosis.

**Figure 3:**
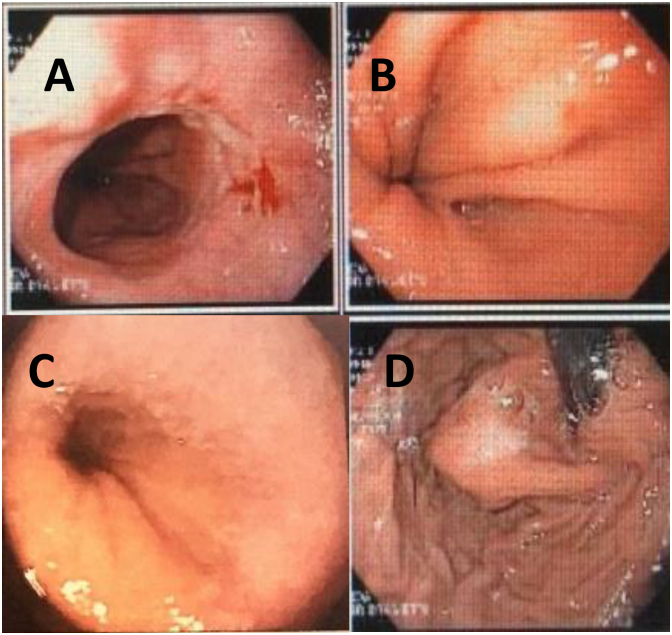
Endoscopic evaluation: (A) Erosive esophagitis, small hiatal hernia after asymmetric Nissen fundoplication; (B) Small type I hiatal hernia; (C) Postoperative endoscopy without esophagitis; (D) Fundoplication.

Postoperatively, wrap stricture, intrathoracic wrap, and twisted fundoplication generating a bilobed stomach were observed in patients who underwent Nissen procedure. Later, a second reoperation was indicated for these patients (Figures 2 and 3). The explanation for this finding is due to difficulty performing the optimal redo fundoplication, in part due to false recognition and localization of anatomical landmarks of EGJ and failure of fundoplication. Patients with dysphagia received endoscopic dilatation, which resulted in improved in symptoms.

Esophagitis recurrence was observed in 23.1% of patients in Group A (redo fundoplication alone). In contrast, in the patients who received redo Nissen or Toupet procedure combined with Roux-en-Y distal gastrectomy, esophagitis was found to be improved significantly after surgery (Table 4). After performing fundoplication alone, persistence of the long segment of BE (C4M7, Praga classification), hiatal hernia, and esophagitis was observed, resulting in the need for second reoperation. Table 5 shows the manometry and 24-h pH monitoring evaluation before and after surgery. Regarding the esophageal motility, a significant difference between the three groups was irrelevant. In Group C, despite the high percentage of patients with incompetent LES, the abnormal acid reflux improved significantly after surgery. In the conversion to Toupet fundoplication with Roux-en-Y distal gastrectomy (Group C) procedure, 23 and 2 (8.7%) patients had pathologic acid reflux before and after the procedure, respectively.

**Table 4:** Endoscopic findings before and after laparoscopic reoperation for the treatment of failed primary Nissen fundoplication.

			A (n=26)	B (n=29)	C (n=23)
Endoscopic evaluation					
	Esophagitis	Pre-operative	17 (65.4%)	13 (44.8%)	5 (21.7%)
		Postoperative	6 (23.1%)*	2 (6.9%)*	0
	BE with esophagitis	Pre-operative	9 (34.6%)	16 (55.2%)	10 (43.5%)
		Postoperative	4 (19.2%)***	6 (20.6%)**	(17.4%)**
	Esophagitis with HH	Pre-operative	0	3	8
		Postoperative	3***	0	0
Reoperated due to refailure			7 (26%)***	0	0

* Medical treatment with esomeprazole;

** Persisted Barrett esophagus without esophagitis submitted to complementary radiofrequency ablation

*** Second reoperation: five to conversion to redo Nissen with distal gastrectomy and two to Toupet with distal gastrectomy; BE: Barrett esophagus; HH: hiatus hernia.

**Table 5:** Functional studies: manometry and 24-h pH monitoring before and after laparoscopic reoperations for the treatment of failed primary Nissen fundoplication.

Manometry			A (n=26)	B (n=29)	C (n=23)
	Laparoscopic Nissen fundoplication pressure (mmHg)	Pre-operative	6.1±2.7	10.1±2.6	7.88±2.7
		Postoperative	11.1±3.8	15.5±1.3	10.5±3.36
	Total length (cm)	Pre-operative	2.5±0.5	3.9±0.2	3.07±0.61
		Postoperative	2.8±0.8	4.05±1.1	3.57±0.53
	Abdominal length (cm)	Pre-operative	0.0	0.3±0.2	0.1±0.2
		Postoperative*	1.8±0.4	1.8±0.4	1.14±0.69
	Number of patients with incompetent laparoscopic Nissen fundoplication	Pre-operative	21 (82%)	29 (100%)	23 (100%)
		Postoperative	9 (34.6%)	5 (17.2%)	6 (26%)
	Amplitude distal waves (mmHg)	Pre-operative	105±17.1	117±18.9	122.4±28.4
		Postoperative	125±20.3	129±25.2	138.9±28.8
	Peristaltic waves (mean) (%)	Pre-operative	43	88	94
		Postoperative	72	94	98
	Number of patients with ineffective motility	Pre-operative	12 (46.1%)	8 (27.5%)	3 (13%)
		Postoperative	6 (23.1%)	5 (17.2%)	1 (4.3%)
24-h pH monitoring					
	Time pH<4 (%)	Pre-operative	25.8±4.9	21.5±4.1	19.0±6.62
		Postoperative#	7.9±3.4	3.6±2.5	4.3±4.04
	DeMeester’s score	Pre-operative	85.3±23.6	69.8±3.4	44.8±21.8
		Postoperative#	23.1±6.9	10.3±4.1#	11.9±5.14
	Number of patients with pathologic acid reflux	Pre-operative	26 (100%)	29 (100%)	23 (100%)
		Postoperative	14 (53.8%)	3 (10.1%)	2 (8.6%)

* p<0.006

# p<0.01.

## DISCUSSION

The gastroesophageal reflux symptoms, retrosternal pain, and dysphagia were reported in 18–61% of patients after Nissen fundoplication, and 4.5–18% of patients require reoperation^4,14,19,24,29,40^.

The etiologies for failure are anatomic causes, such as slipped fundoplication, asymmetric or disrupted wrap, and herniated fundoplication, resulting in the appearance of gastroesophageal reflux symptoms^5,21,22,40^.

The options for surgery include redo fundoplication alone with hiatal hernia repair if needed, and conversion to distal gastrectomy with Roux-en-Y gastrojejunostomy either alone or in combination with redo fundoplication. The decision is not easy because there are many factors to take in account, such as obesity, grade of esophagitis, presence of BE, type of anatomic abnormality, presence of stricture or hiatal hernia, type of surgery performed before, number of reoperations performed, gastric emptying, and the presence of acid and bile reflux. Consequently, the ideal treatment option is not clear^3,11,13,25,38,46,47^.

Redo fundoplication is the first and most frequently performed technique (in 89% of cases), although it has long operative time, high rate of postoperative complications, and longer hospital stay associated with very variable success. The reported satisfactory outcome after re-operative fundoplication was as low as 50% (range 42–94%), and the possibility of a new failure after redo Nissen fundoplication is observed in 40% of cases. Even worst results have been reported after the second reoperation in terms of incomplete relief of symptoms (12–50%), with the satisfaction rate being about 42%^18,34,35,40^.

Which of the procedures is the best: redo fundoplication, Nissen, or Toupet redo fundoplication? There was no marked difference in the recurrence rate between the two procedures with equivalent satisfaction rate. Nevertheless, dysphagia early after operation has been observed at a higher frequency with the Nissen procedure (although this appears to resolve, in most cases), compared to the Toupet procedure. LNF patients had higher Eckardt dysphagia scores 1 year after surgery compared to LTF patients, but this difference is not found at 3 or 5 years postoperatively. Comparison of laparoscopic 270° posterior partial fundoplication versus total fundoplication suggests that although LTF and LNF could be recommended for the treatment of GERD, LTF might be superior by inducing less dysphagia^27^. In contrast, Toupet procedure may not be as durable^26,29,34,35^. Ottignon et al.^39^ reported the presence of gastroesophageal reflux symptoms in 17% of patients.

Recent studies have suggested to perform distal gastrectomy plus a redo fundoplication in order to correct the possible anatomical alterations of the previous fundoplication^2,5,17,36,37,43,45,48^. In our opinion, it is necessary to dissect the previous fundoplication in order to correct anatomic abnormalities causing symptoms, mainly dysphagia and pain. If this maneuver is not performed, persistence of these symptoms can occur^11,14,25,35,36,44,45,48^. We observed that resection of distal stomach is more complex, due to associated postoperative complications; however, we are expertise with this technique, even when open laparotomy and laparoscopic approach are widely adopted^1,6–10,44^.

In this report, we present the results comparing Nissen versus Toupet combined with distal gastrectomy. The reason for the change in our surgical strategy is based on the very known risk of dysphagia following Nissen.

Although the Toupet technique reduces the risk of postoperative dysphagia in patients with indication for revision surgery after Nissen, it does not ensure better LES pressure, which could result in recurrence of reflux and esophagitis. Therefore, we postulate that adding a distal subtotal gastrectomy could possibly avoid both acid and bile refluxes^16,21,31–34,41,44^.

The other advantage of distal gastrectomy is its ability to stop biliary reflux. When combined with ablation of metaplastic epithelium, the risk of dysplastic changes is reduced. In case of the eventual need for esophagectomy, it is possible to indicate colon interposition.

The limitations of this study are as follows:

- the number of patients included in each group is small, but it is representative;
- only early- and mid-term follow-up; and
- it is not a randomized study, the procedures were chosen according to the clinical presentation.

The strengths of this study are as follows:

- prospective study;
- complete follow-up (100%);
- all patients have objective evaluation; and
- it is the first comparative study presenting results of three different procedures and combination of Nissen versus Toupet procedure with distal gastrectomy and Roux-en-Y gastrojejunostomy.

## CONCLUSION

Nissen and Toupet procedures combined with Roux-en-Y distal gastrectomy are safe and effective for the management of failed Nissen fundoplication. However, Toupet technique is preferable for patients suffering from mainly dysphagia and pain.
